# Systematic Construction and Validation of an RNA-Binding Protein-Associated Model for Prognosis Prediction in Hepatocellular Carcinoma

**DOI:** 10.3389/fonc.2020.597996

**Published:** 2021-01-26

**Authors:** Siyuan Tian, Jingyi Liu, Keshuai Sun, Yansheng Liu, Jiahao Yu, Shuoyi Ma, Miao Zhang, Gui Jia, Xia Zhou, Yulong Shang, Ying Han

**Affiliations:** ^1^ State Key Laboratory of Cancer Biology and National Clinical Research Center for Digestive Diseases, Xijing Hospital of Digestive Diseases, The Fourth Military Medical University, Xi’an, China; ^2^ Department of Radiation Oncology, Xijing Hospital, The Fourth Military Medical University, Xi’an, China

**Keywords:** prognostic model, survival analysis, hepatocellular carcinoma, RNA-binding proteins, The Cancer Genome Atlas (TCGA)

## Abstract

**Background:**

Evidence from prevailing studies show that hepatocellular carcinoma (HCC) is among the top cancers with high mortality globally. Gene regulation at post-transcriptional level orchestrated by RNA-binding proteins (RBPs) is an important mechanism that modifies various biological behaviors of HCC. Currently, it is not fully understood how RBPs affects the prognosis of HCC. In this study, we aimed to construct and validate an RBP-related model to predict the prognosis of HCC patients.

**Methods:**

Differently expressed RBPs were identified in HCC patients based on the GSE54236 dataset from the Gene Expression Omnibus (GEO) database. Integrative bioinformatics analyses were performed to select hub genes. Gene expression patterns were validated in The Cancer Genome Atlas (TCGA) database, after which univariate and multivariate Cox regression analyses, as well as Kaplan-Meier analysis were performed to develop a prognostic model. Then, the performance of the prognostic model was assessed using receiver operating characteristic (ROC) curves and clinicopathological correlation analysis. Moreover, data from the International Cancer Genome Consortium (ICGC) database were used for external validation. Finally, a nomogram combining clinicopathological parameters and prognostic model was established for the individual prediction of survival probability.

**Results:**

The prognostic risk model was finally constructed based on two RBPs (BOP1 and EZH2), facilitating risk-stratification of HCC patients. Survival was markedly higher in the low-risk group relative to the high-risk group. Moreover, higher risk score was associated with advanced pathological grade and late clinical stage. Besides, the risk score was found to be an independent prognosis factor based on multivariate analysis. Nomogram including the risk score and clinical stage proved to perform better in predicting patient prognosis.

**Conclusions:**

The RBP-related prognostic model established in this study may function as a prognostic indicator for HCC, which could provide evidence for clinical decision making.

## Introduction

Hepatocellular carcinoma (HCC) is classified as one of the most prevalent cancers globally, posing a serious threat to human health. According to the global cancer statistics, there were nearly 782,000 cancer-associated deaths and 841,000 new cases of HCC, ranking fourth and sixth in all tumors, respectively (1). Although much progress have been made in the diagnosis and treatment of HCC, 5-year survival rate has not been improved largely due to high rates of recurrence and metastasis (2, 3). The occurrence and progression of HCC are driven by multiple processes affected by both genetic and environmental factors (4). In recent years, accumulating evidences have suggested that post-transcriptional modifications play an important role in the malignant phenotype of tumors, and these changes are strongly linked to the clinical course of patients (5–7). Thus, it’s of great significance to systematically explore relevant biological biomarkers for HCC management.

RNA-binding protein (RBP) is a general term for the group of proteins that exert its function by binding to RNA specifically, such as RNA maturation, transport, localization and translation. To date, human genome-wide screening has identified 1,542 RBP genes, accounting for 7.5% of all protein-coding genes (8). With few exceptions, most of RNAs are required to form RNA-protein complex to perform specific biological functions (9). In this process, RBPs exert essential role, especially in gene expression and the maintenance of genome integrity (10, 11). While the significance of post-transcriptional regulation to tumor initiation and progression has been recognized, the role of RBPs in HCC is still rudimentary. Several studies have demonstrated the aberrant expression of RBPs in HCC (6, 12–14). For instance, RBP RPS3 was markedly up regulated in HCC tumor tissues and served as a critical tumor-promoting factor *via* up-regulating SIRT1. Targeting against the RPS3/SIRT1 pathway holds much promise as a target for therapeutic exploitation for the treatment of HCC (12). RBP eIF3C has been reported to promote cell growth *in vitro* and tumorigenicity *in vivo* (14). Furthermore, Yang et al. reported that RBP MEX3A could independently reflect the clinical course of HCC, and overexpression of MEX3A predicted poor prognosis (13). However, large-scale prognostic signature based on RBP genes in HCC has rarely been investigated. Therefore, a systematic study to explore prognosis-related RBP genes will be helpful to understand their roles in the initiation and progression of HCC, and will also have great value in guiding decision-making.

In this study, RBP genes expression data was derived from The Cancer Genome Atlas (TCGA), International Cancer Genome Consortium (ICGC) and Gene Expression Omnibus (GEO) databases to develop and validate a prognostic model for long-term prognostic prediction of HCC patients. To make full use of the complementary significance of molecular expression and clinical features, we combined the prognostic model with clinical parameters to construct a comprehensive prognostic nomogram, further improving the predictive power.

## Materials and Methods

### Data Source

HCC GeneChip data (GSE 54236) was derived from the National Center for Biorechnology Information (NCBI) GEO DataSets. GSE 54236 was prepared from the Agilent GPL 6480 platform (Agilent-014850 Whole Human Genome Microarray 4×44K G4112F) including 81 human HCC tissue and 80 non-tumor tissue samples. Besides, we downloaded the level-three transcriptome RNA-sequencing data of LIHC from 344 samples with corresponding clinical data in the TCGA database for establishing a prognostic model. Meanwhile, to test the accuracy of the model, a validation set including clinical information and RNA-seq data was also downloaded from the ICGC database (LIRI-JP cohort). The detailed clinical information of TCGA dataset and ICGC dataset was shown in **Supplementary Table 1**. All data used in this study were freely available online.

### Identification of Differently Expressed RNA-Binding Proteins

The GEO2R, an online analysis tool in GEO database, was used to identify differently expressed genes (DEGs) in HCC tissue samples and adjacent normal tissue samples (15). The log2^|FC|^≥0.5 and adjusted P-value ≤0.05 were set as the cut-off criterion. DEGs were then processed to generate a volcano plot with GraphPad Prism 7 Software. Next, we used the Venn diagram webtool to determine the overlapping between the DEGs and 1542 Human RNA-binding proteins for further analysis. Heat map of the differently expressed RBPs was plotted by the online software NetworkAnalyst (16).

### Gene Ontology and Kyoto Encyclopedia of Genes and Genomes Enrichment Analyses

To further investigate the potential function and molecular mechanisms involved by these differently expressed RBPs, Gene Ontology (GO) and Kyoto Encyclopedia of Genes and Genomes (KEGG) enrichment analyses were conducted using the clusterProfiler package in R (version. 4.0). The GO terms covered three biological aspects including molecular function (MF), cellular component (CC) and biological process (BP). *P* values of <0.05 were regarded as statistically significant.

### Protein–Protein Interaction Network Construction and Hub Genes Analysis

Protein-Protein interactions (PPI) were assessed using the online STRING database (https://string-db.org/). The protein-protein pairs of differently expressed RBPs meeting the criteria of combined score > 0.4 were selected and displayed by the Cytoscape software (version 3.7.2). Then, cytoHubba, a plug-in of Cytoscape, was used to screen out the gene with the highest degrees of connectivity. The top ten genes were considered the hub genes and hence used in subsequently analyses.

### Screening and Analysis of Prognosis-Related RBPs

The UALCAN database (http://ualcan.path.uab.edu/) is an open network resource for data-mining, primarily based on the respective cancer data in TCGA database. Herein, mRNA levels of hub genes in normal and HCC tissues were compared in the UALCAN database. Besides, the correlation between gene-expression levels and tumor grades were further analyzed. The protein levels of the hub genes were also compared between normal and HCC tissues by immunohistochemical image in Human Protein Atlas (HPA) database (http://www.proteinatlas.org). To further explore the prognostic value of hub RBPs, we conducted a survival analysis using Kaplan-Meier plotter (http://www.kmplot.com). Survival curves and corresponding parameters could be obtained at the website. Finally, genetic alteration data of all prognosis-related hub genes were downloaded and visualized in the form of mutation profiles from the cBioPortal (http://www.cbioportal.org).

### Construction and Validation of an RNA-Binding Protein-Associated Prognostic Model

Univariate Cox regression analysis was performed using the “survival” package in R software to identify prognostic factors from above prognosis-related RBPs. Variables with P < 0.05 in univariate analysis were further included in the stepwise multivariate analysis. A predictive model was then designed based on the independent prognostic factors. The following formula was used to determine the risk score: Risk score = β1*Exp1+β2*Exp2+βi*Expi (β, regression coefficient; Exp, prognostic factors). Afterwards, we sub-classified patients into low and high risk groups according to the median risk score. The difference of survival status between the two groups was assessed by Kaplan-Meier curves. Meanwhile, the “time ROC” package was used to establish a ROC curve to assess the prognostic value of the model by calculating the area under the curve (AUC). Finally, the prognostic model was externally validated using the ICGC dataset to test its stability.

### Establishing a Nomogram for Prognostic Risk Assessment

To ascertain if the constructed model performed was independently of clinical features, multivariate analysis was conducted using the Cox regression model. Then, we established a nomogram integrating the factors with independent prognostic value, which could provide quantitative methods to predict survival probabilities of HCC patients. The prognostic accuracy of the nomogram was further evaluated using the ROC curve analysis and calibration plots.

## Results

### Differently Expressed RNA-Binding Proteins in Hepatocellular Carcinoma

**Figure 1:**
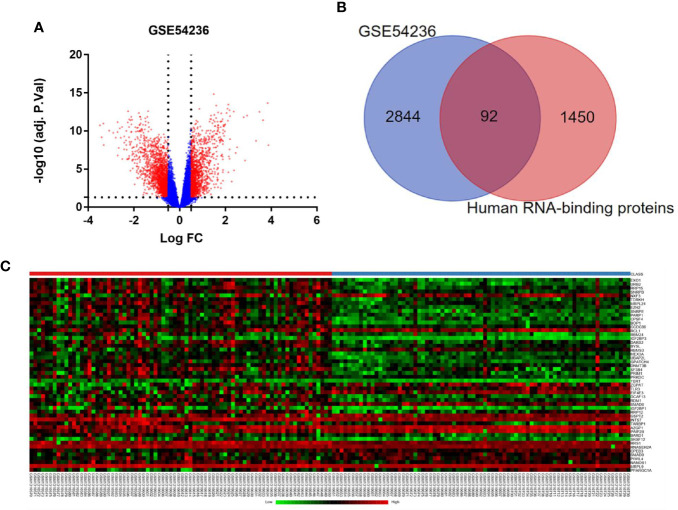
Differently expressed RBPs in hepatocellular carcinoma. **(A)** Volcano plots of DEGs from GSE 54236. Red plots represent genes with log^|FC|^ ≥ 0.5 and adjusted P-value ≤ 0.05. **(B)** Venn diagram of the overlap between the DEGs from GSE 54236 and candidate RBP genes catalog. **(C)** Heatmap of top 50 differently expressed RBP genes.

A flowchart of the study is provided as **Supplementary Figure 1**. After removing duplicate samples, 78 HCC tissues and 77 adjacent non-tumor tissues were processed for data-mining by GEO2R, and 2,936 differently expressed genes (DEGs) meeting the screening criteria were identified (**Figure 1A**). We then used Venn diagram to show the intersection between these DEGs and candidate RBP genes catalog. Finally, a total of 92 RBPs were found, among which 27 were down-regulated and 65 were up-regulated (**Figure 1B**). Heatmap of the top 50 differently expressed RBPs were plotted in **Figure 1C**.

### Functional Enrichment of Differently Expressed RNA-Binding Proteins

**Figure 2:**
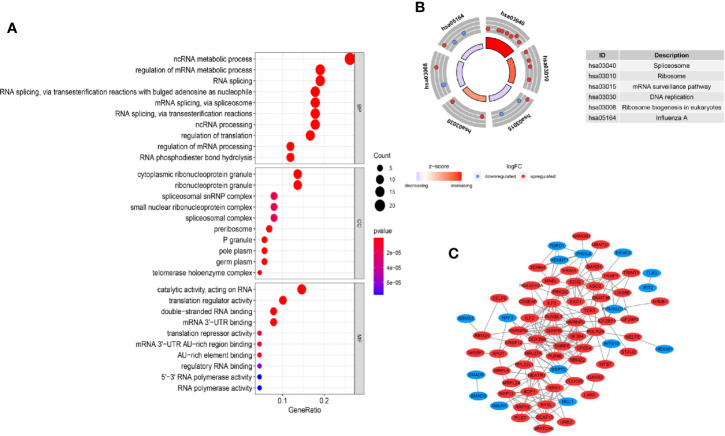
GO, KEGG enrichment analysis and PPI network. **(A)** GO analysis of differently expressed RBP genes. MF: molecular function; CC: cellular component, BP: biological process; **(B)** KEGG analysis of differently expressed RBP genes. **(C)** PPI network of differently expressed RBP genes.

GO enrichment and KEGG pathway analyses were applied to clarify the gene function of differently expressed RBPs and signaling pathways involved. The GO terms were clustered into 3 major GO ontologies: BP, CC and MF. As shown in **Figure 2A**, differently expressed RBPs were mostly enriched in BP: including ncRNA metabolic process, regulation of mRNA metabolic process and RNA splicing. For CC, DEGs were mostly enriched in cytoplasmic ribonucleoprotein granule and ribonucleoprotein granule. Molecule Function analysis indicated that the DEGs significantly participated in double-stranded RNA binding, mRNA 3’-UTR binding and translation repressor activity. Moreover, for the KEGG pathway analysis, our results revealed that DEGs were mostly enriched in Spliceosome, Ribosome and mRNA surveillance pathway (**Figure 2B**).

### Protein–Protein Interaction Construction and Hub Genes Identification

*In vivo*, protein–protein interaction is an important way to exert their biological function. A PPI network of differently expressed RBPs was established based on the STRING database and visualized using Cytoscape Software. The network composed of 74 nodes and 214 links, as shown in **Figure 2C**. Next, applying the cytoHubba plug-in, top ten genes were selected from the PPI network according to their connectivity degrees. These hub genes including POLR2K, HNRNPM, SNRPE, DDX39A, RRS, HNRNPU, BOP1, SNRPB, EZH2, and ILF3 were all up-regulated. The gene description and connectivity degrees of these hub genes were summarized in **Table 1**.

**Table 1:** The top 10 hub genes with the highest degree of connectivity in the PPI network.

Gene symbol	Gene description	Degree
POLR2K	RNA Polymerase II Subunit K	16
HNRNPM	Heterogeneous Nuclear Ribonucleoprotein M	15
SNRPE	Small Nuclear Ribonucleoprotein Polypeptide E	15
DDX39A	DExD-Box Helicase 39A	14
RRS1	Ribosome Biogenesis Regulator 1 Homolog	14
HNRNPU	Heterogeneous Nuclear Ribonucleoprotein U	14
BOP1	BOP1 Ribosomal Biogenesis Factor	13
SNRPB	Small Nuclear Ribonucleoprotein Polypeptides B And B1	13
EZH2	Enhancer Of Zeste 2 Polycomb Repressive Complex 2 Subunit	11
ILF3	Interleukin Enhancer Binding Factor 3	11

### Verification of the Hub Genes

**Figure 3:**
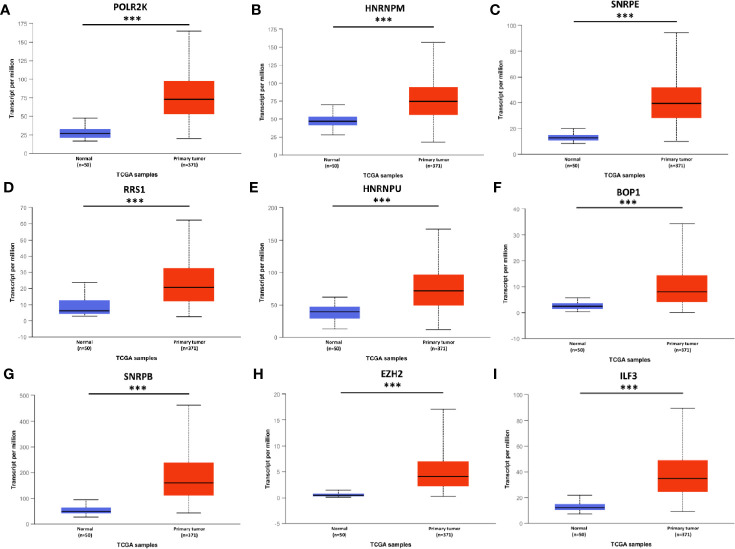
mRNA expression of hub genes in HCC tissues and adjacent normal tissues. All these hub genes were highly expressed in tumor tissues **(A–I)**. *** P < 0.001.

**Figure 4:**
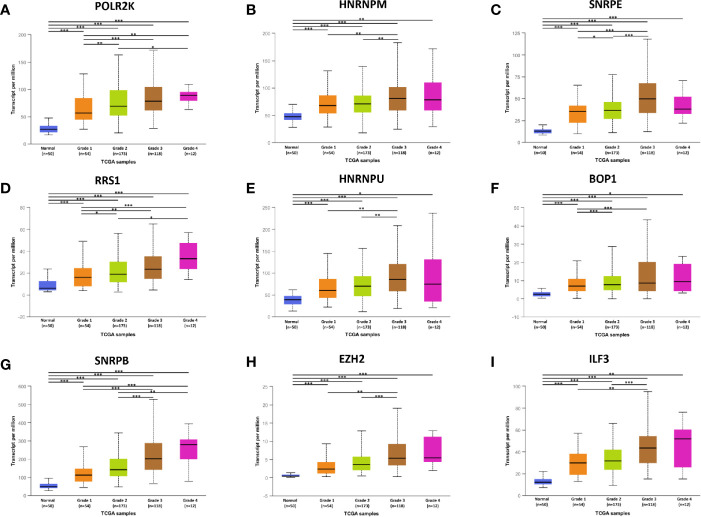
The relationship between hub genes expression and tumor grades in HCC. *P < 0.05, **P < 0.01, ***P < 0.001.

To confirm the results, above hub genes were verified in the UALCAN database including gene expression patterns and correlation with clinicopathological parameters. The results showed that a total of nine hub genes were matched in the UALCAN database and their expression were significant elevated in tumor tissues (**Figure 3**). Besides, hub gene expression was positively correlated with tumor pathological grade (**Figure 4**).

After detecting the hub genes expression at mRNA level, we also compared their protein expression in normal and liver cancer tissues using immunohistochemical results obtained from HPA database (**Supplementary Figure 2**). We found that RRS1, HNRNPU, SNRPB, EZH2, and ILF3 protein expression levels were increased in HCC tissues than normal tissues, whereas changes for HNRNPM and BOP1 were not significant. Additionally, POLR2K and SNRPE were not detected in either tumor or non-tumor tissues.

### Prognosis-Related RNA-Binding Protein Selection and Genetic Alteration Analysis

**Figure 5:**
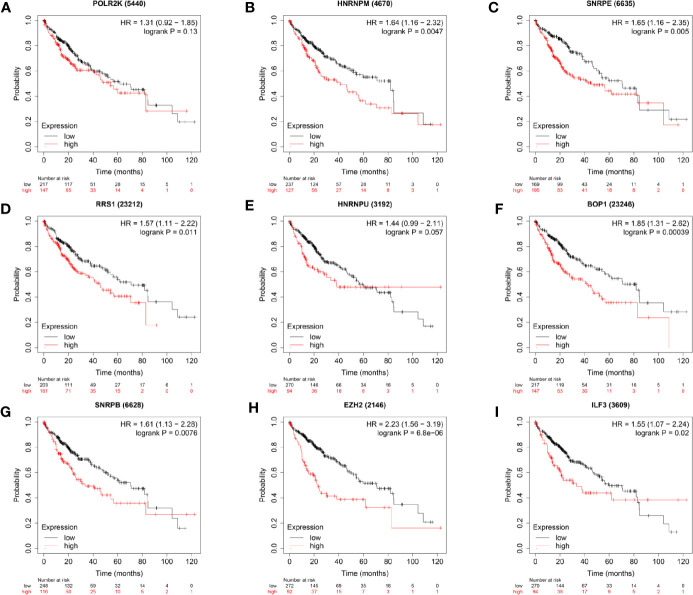
Survival analyses of the hub genes in patients with HCC (Kaplan–Meier Plotter). P < 0.05 was considered statistically significant.

Considering above results, expression of hub RBPs were strongly correlated with the degree of tumor malignancy. Thus, the association of the hub RBPs expression with the prognosis of HCC patients was explored in the Kaplan-Meier Plotter database. Survival data from 364 HCC patients were available for analysis. As shown in **Figure 5**, higher expression of HNRNPM (HR = 1.64, 95% CI: 1.16–2.32, and P = 0.0047), SNRPE (HR = 1.65, 95% CI: 1.16–2.35, and P = 0.005), RRS1 (HR = 1.57, 95% CI: 1.11–2.22, and P = 0.011), BOP1 (HR = 1.85, 95% CI: 1.31–2.62, and P = 0.00039), SNRPB (HR = 1.61, 95% CI: 1.13–2.28, and P = 0.0076), EZH2 (HR = 2.23, 95% CI: 1.56–3.19, and P = 6.8×10^−6^), and ILF3 (HR = 1.55, 95% CI: 1.07–2.24, and P = 0.02) were significantly associated with reduced survival period of patients with liver cancer. However, high expression of POLR2K or HNRNPU didn’t show significant prognostic value.

Next, gene mutations of the seven prognosis-related RBPs were analyzed in cBioPortal database. The results showed that, out of the 345 HCC patients, the seven RBP genes showed altered expression patterns in 214 (62%) samples. Moreover, we found that the mutation rate of BOP1 was 38%, ranking first in all prognosis-related RBPs (**Supplementary Figure 3**).

### Construction and Validation of the Prognostic Model

After identifying the seven prognosis-related RBPs, we then tried to evaluate their independent prognostic value as biomarkers for overall survival (OS) of patients with HCC. Cox regression analysis was performed based on expression data of prognosis-related RBP genes. Univariate analysis showed that all seven RBPs were the influencing factors for unfavorable survival of liver cancer patients. In the multivariate analysis, we found that high expression of BOP1 (HR = 1.259, 95%CI: 1.064–1.489, and *p* = 0.007) and high expression of EZH2 (HR = 1.801, 95%CI: 1.409–2.303, and *p* < 0.001) were related to poor prognosis (**Table 2**). Then, the risk score formula based on regression coefficients of the above two genes can be obtained as follow:

**Table 2:** Association between genes expression and overall survival in HCC patients.

Gene symbol	HR(95%CI)	P-Value
A.		
HNRNPM	2.025(1.401–2.925)	0.000
SNRPE	1.287(1.026–1.615)	0.029
RRS1	1.242(1.014–1.521)	0.036
BOP1	1.321(1.121–1.558)	0.000
SNRPB	1.498(1.201–1.868)	0.000
EZH2	1.864(1.465–2.373)	0.000
ILF3	1.575(1.157–2.144)	0.004
B.		
BOP1	1.259(1.064–1.489)	0.007
EZH2	1.801(1.409–2.303)	0.000

(A) Univariate regression analysis. (B) Multivariate regression analysis.

Risk score = 0.2304 * BOP1 + 0.5885 * EZH2.

**Figure 6:**
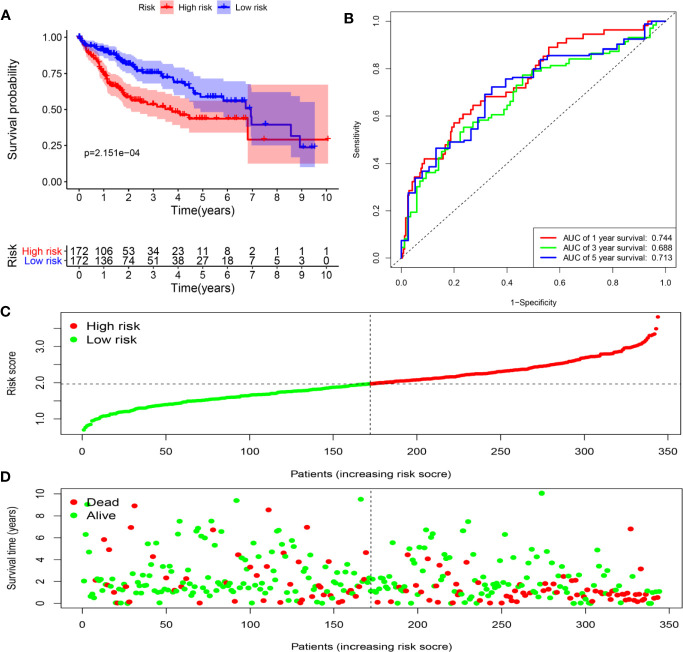
Determination of risk scores by the prognostic model in the TCGA dataset. **(A)** Survival analysis of high- and low-risk groups. **(B)** ROC curves analysis of the prognostic model. **(C)** The risk score distribution of HCC patients. **(D)** The survival status of patients in the TCGA dataset.

**Figure 7:**
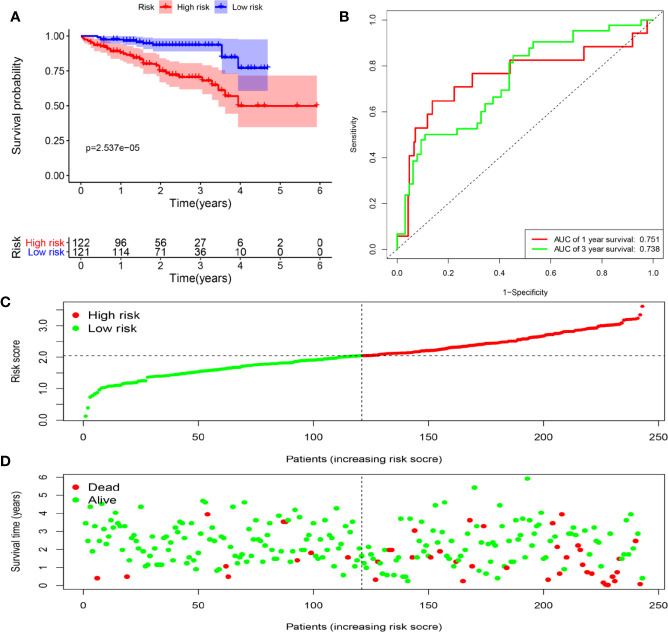
Determination of risk scores by the prognostic model in the ICGC dataset. **(A)** Survival analysis of high- and low-risk groups. **(B)** ROC curves analysis of the prognostic model. **(C)** The risk score distribution of HCC patients. **(D)** The survival status of patients in the ICGC dataset.

To assess the ability of the model to predict prognosis, survival analysis was conducted. A total of 344 HCC patients were sub-classified into low and high groups based on the medium risk score value. In the TCGA dataset, the OS rate of patients in the high-risk group was markedly lower compared to that of patients in the low-risk group (**Figure 6A**). Time-dependent ROC curves at 1, 3 and 5 years were plotted, and the AUC at 1, 3 and 5 years survival was 0.744, 0.688 and 0.713, respectively (**Figure 6B**). Besides, the distribution of patients’ risk score, survival time and survival status were shown in **Figures 6C, D**. Then, the stability of this model was examined in an ICGC-JP dataset. Consistent with the TCGA dataset, patients in the high-risk group exhibited a substantially worse outcome (**Figure 7A**). We also applied ROC curve analysis to evaluate the performance of the prognostic model, and the AUC value at 1 and 3 years was 0.751 and 0.738, respectively (**Figure 7B**). Risk score and survival information were presented in **Figures 7C, D**. All these results suggested good accuracy and stability of this model. Compared with OS, disease-free survival (DFS) and disease-specific survival (DSS) can reflect clinical benefits more specifically. To examine whether the prognostic model is also related to DFS and DSS, these two prognostic indicators were used as clinical endpoints for survival analysis. Compared with the low-risk group, the DFS was significantly reduced in high-risk patients (**Supplementary Figures 4A, B**). The area under the curve (AUC) of time-dependent ROC curves at 1, 3, and 5 years was 0.710, 0.582 and 0.747, respectively (**Supplementary Figure 4C**). Similar conclusions could also be reached for DSS (**Supplementary Figures 4D–F**).

### Clinical Utility of the Established Prognostic Model

**Figure 8:**
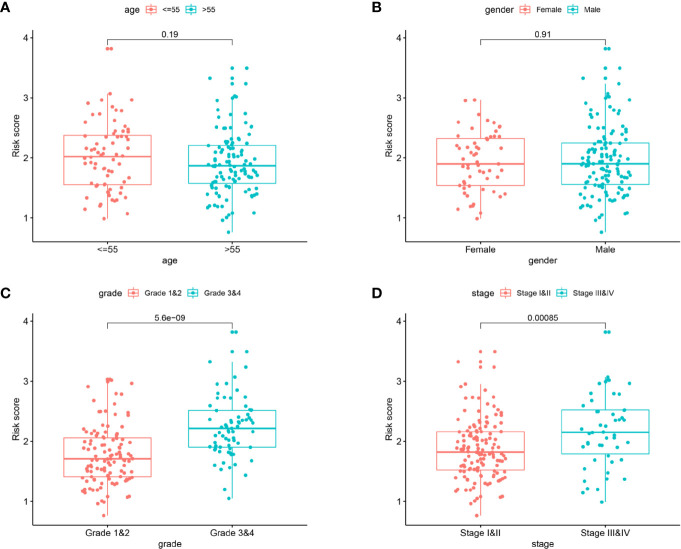
The clinicopathological significance of the prognostic model. Age **(A)** and gender **(B)** were not associated with the risk score. Advanced pathological grade **(C)** and late clinical stage **(D)** were found to be associated with a high risk score.

Considering the risk score were closely related to the survival of patients, we subsequently analyzed the relationships between the prognostic model and clinical characteristics. As shown in **Figure 8**, the risk score was remarkably higher in advanced pathological grade (P < 0.001) and late clinical stage (P < 0.001). No significant difference was found in age > 55 versus age ≤ 55 (P = 0.19), or female versus male (P = 0.91).

### Construction of a Nomogram for Clinical Practice

**Figure 9:**
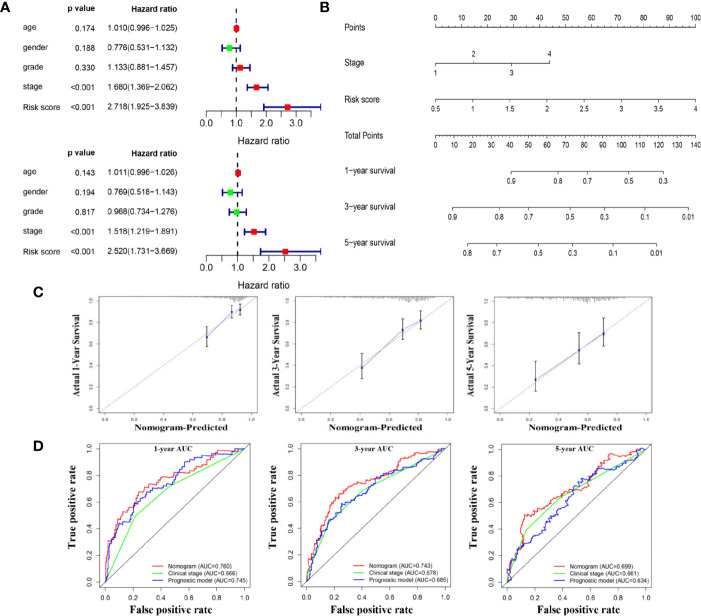
A nomogram for overall survival prediction for HCC patients. **(A)** Univariate and multivariate Cox analyses for the prognostic model and clinical parameters. **(B)**. A nomogram to predict OS at 1 year, 3 years, and 5 years of patients with HCC. **(C)**. Calibration plots showing the prediction of OS at 1 year, 3 years, and 5 years by the nomogram **(D)**. Comparison of time-dependent ROC curves among nomogram, clinical stage and the prognostic model.

Further analysis revealed that risk score and pathological stage were independent prognostic factors for OS (**Figure 9A**). Then, the R software was used to integrate these two variables to construct a nomogram-plot (**Figure 9B**). By drawing vertical line to the points axis, we could calculate the sum of the scores of all variables, and the corresponding probability is the survival probability of patients. Calibration curves in TCGA dataset suggested that the predicted values of the nomogram matched well with the actual survival probability of the patients (**Figure 9C**). We also compared the discrimination performance among the clinical stage, risk score and the nomogram. As shown in **Figure 9D**, the nomogram exhibited the largest AUC value, indicating good clinical application value. Moreover, we also constructed a nomogram for DFS prediction, and confirmed the model with good predictive power (**Supplementary Figure 5**).

## Discussion

HCC is a highly heterogeneous cancer with high mortality and different clinical outcomes. So far, there have been no solidly established prognostic biomarkers for risk prediction in clinical practice. Hence, the search for accurate, reliable and simple prognostic biomarkers is especially significant for HCC patients (17). Being important epigenetic factors involved in post-transcriptional regulation, RBPs participate in the initiation and development of various tumors including HCC, revealing their potential to serve as prognostic biomarkers (18–21). In studies by Feng et al. (22), RBM43 was identified as a tumor suppressor by inhibiting carcinogenesis and tumor growth, and its low expression was negatively correlated with HCC prognosis. Similarly, low expression of SORBS2 was regarded as an adverse prognostic marker (23, 24). Moreover, several other studies revealed that overexpression of certain RBPs exhibited positive correlations with the recurrence and poor prognosis of HCC patients (13, 25, 26). Although these studies have explored the possible functions of certain RBPs and confirmed their prognostic value, further study is required to investigate the clinical value of RBPs in HCC, comprehensively and systematically. Herein, we mined expression profiles of RBP from GEO, TCGA and ICGC datasets and aimed to explore the impact of RBP gene expression level on the prognosis of HCC patients.

A total of 92 differently expressed RBP genes between HCC and non-tumor tissues were selected from the GEO dataset, which were mainly enriched in the processes involved in the regulation of RNA. Through the PPI network construction, ten hub genes exhibiting the highest degrees were identified in the network. After validation in the TCGA database, survival analyses, univariate, and multivariate Cox regression analyses were performed to screen out survival-related RBP genes. Based on two survival-related RBP genes, a prognostic model was finally constructed for prediction of clinical outcomes. Patients with higher risk scores showed worse overall survival, which was confirmed in both TCGA and ICGC databases. To specifically reflect clinical benefits, DFS and DSS were additionally used as the clinical endpoints, and this model still exhibited good predictive capability. Furthermore, multivariate analysis revealed that the prognostic model could be used as an independent prognostic biomarker for HCC patients. A nomogram combined pathological stage with prognostic model will provide a more precise individualized prediction.

Two RBP genes identified in this study were previously reported to function as important regulators driving the initiation and progression of diverse tumors. The block of proliferation (BOP1) gene, located on chromosome 8q24.3, belongs to the WD40 protein family (27). As part of the PES1-BOP1-WDR12 (PeBow) complex, the BOP1 gene is mainly involved in 60S ribosome maturation process (28). Abnormal expression of BOP1 modifies various aspects of colorectal cancer and melanoma (29, 30). In colorectal cancer cells, BOP1 was identified as a target gene of the Wnt/β-catenin pathway. Overexpression of BOP1 promoted cell migration, epithelial-mesenchymal-transition and experimental metastasis of CRC cells as a function of its crosstalk with the JNK signaling pathway and downstream genes (29). In melanoma, Gupta et al. reported that BOP1 knockdown played a crucial role in the acquisition of BRAF kinase inhibitors resistance by targeting MAPK phosphatases DUSP4 and DUSP6 (30). Recently, it has been demonstrated that the BOP1 gene was overexpressed in HCC tissues, and showed strong association with microvascular invasion and advanced disease stage. Moreover, evidence from *in vitro* studies suggested that BOP1 gene exerted oncogenic effects by inducing EMT and actin cytoskeleton remodeling (31). Similar roles of BOP1 gene in HCC progression were also found in the present study. Our results showed that the expression level of BOP1 was significantly higher in HCC tissues compared with paracancerous tissues. Besides, high expression of BOP1 was associated with tumor grade and poor survival, acting as an independent prognostic factor for HCC patients.

The enhancer of zeste homolog2 (EZH2) gene encodes the histone-lysine N-methyltransferase enzyme, participating in the silencing of many tumor suppressor genes (32, 33). As expected, over-expression of EZH2 was found in a variety of malignancies, such as prostate cancers (34), breast cancers (35) and gastric cancers (36). Studies from Xu et al. showed that EZH2 was over-expressed in HCC tissues and cell lines, and miR-101 could target EZH2 to inhibit HCC progression and increase chemotherapeutic treatment sensitivity (37). Interestingly, Hibino et al. found that the inhibition of EZH2 by small molecule agents could also activate tumor-suppressor miRNAs to exert antitumor effects in HepG2 cells (38). In recent years, immune checkpoint inhibitors appear to be a promising approach for HCC treatment. Xiao et al. found epigenetic modifications mediated by EZH2 could also affect immunotherapy effect. Mechanistically, EZH2 could negatively regulate the expression of PD-L1 by regulating the promoter H3K27me3 levels of CD274 (39). Herein, the protein and mRNA expression of EZH2 were found significantly higher in HCC tissues, and mRNA expression of EZH2 was highly correlated with tumor grades and prognosis of HCC patients.

With the development of microarray or RNA sequence techniques, several prediction models have been proposed to facilitate the prediction of long-term outcomes of HCC patients. However, some limitations should be considered and addressed in these studies. For instance, Sun et al. constructed a prognostic signature containing 33 immune gene pairs (40). The model incorporating too many variables was not easy to use, which limited its clinical application. Besides, the robustness of predictive models was not given enough attention in the research of Zheng et al. (41). An external validation in different population was needed to test the reliability of predictive models. Moreover, some predictive models did not include relevant clinical parameters, which to some extent weakened the diagnostic effectiveness of the models (42, 43).

Recently, a similar study has been published. In that study, the investigators constructed a gene signature containing 37 gene pairs to predict the overall survival of HCC patients (44). The gene signature in our study contained only two genes, which seemed to be less comprehensive than the former. However, the gene signature in our study still showed good predictive ability and high stability. In contrast, it could be more easy-to-use and cost-effective in clinical practice. The robustness of this prognostic signature was also validated in the ICGC-JP series from the high incidence area of HCC, further extending its applicability across different race groups. Besides, the prognostic signature was confirmed using different study endpoints including OS, DFS and DSS, making the application more convenient and accurate. On the basis of the model, pathological stage was added to establish a nomogram, further enhancing the performance for the prediction of survival. All these findings may provide a better guidance for clinical decision-making of individual therapy.

Meanwhile, some limitations of this study should also be noted. Although we attempted to perform a comprehensive analysis to conduct the retrospective study, further prospective studies are needed to verify the conclusion. In addition, considering the heterogeneity between study populations, it will be necessary to validate the effectiveness and reliability of the model with more patients’ samples from different centers.

## Data Availability Statement

The datasets presented in this study can be found in online repositories. The names of the repository/repositories and accession number(s) can be found below: https://www.ncbi.nlm.nih.gov/, GSE54236 https://cancergenome.nih.gov/, TCGA-LIHC https://dcc.icgc.org/, ICGC-JP.

## Author Contributions

YH, YS, and XZ conceived and designed the experiments. JY, MZ, and GJ collected the data. ST, JL, KS, YL, JY, and SM analyzed the data. ST, JL, KS, and YL drafted the paper. All authors critically reviewed the manuscript. All authors contributed to the article and approved the submitted version.

## Conflict of Interest

The authors declare that the research was conducted in the absence of any commercial or financial relationships that could be construed as a potential conflict of interest.
