# Supplementary material for: Systematic Construction and Validation of an RNA-Binding Protein-Associated Model for Prognosis Prediction in Hepatocellular Carcinoma
Source: Front Oncol. 2021 Jan 26;10:597996. doi: 10.3389/fonc.2020.597996 (PMC7870868; doi:10.3389/fonc.2020.597996)
Supplement: Supplementary file 1 [file Table_1.docx]

**Supplementary Table 1. The detailed clinical information of TCGA dataset and ICGC dataset.**

| **Characteristics** | **TCGA cohort** | **ICGC cohort** |
| --- | --- | --- |
|  | **(N=344)** | **(N=243)** |
| **Age at diagnosis (years)** |  |  |
| ＞55 | 222 (65.0%) | 216 (88.9%) |
| ≤55 | 122 (35.0%) | 27 (11.1%） |
| **Sex** |  |  |
| Male | 234 (68.0%) | 178 (73.3%) |
| Female | 110 (32.0%) | 65 (26.7%) |
| **Stage** |  |  |
| Ⅰ | 170 (49.4%) | 36 (14.8%) |
| Ⅱ | 84 (24.4%) | 105 (43.2%) |
| Ⅲ | 85 (24.7%) | 71 (29.2%) |
| Ⅳ | 5 (1.5%) | 19 (7.8%) |
| Unknown | 0 | 12 (5.0%) |
| **T classfication** |  |  |
| T1 | 171 (49.7%) | NA |
| T2 | 86 (25.0%) | NA |
| T3 | 76 (22.1%) | NA |
| T4 | 10 (2.9%) | NA |
| Unknown | 1 (0.3%) | NA |
| **N classfication** |  |  |
| N0 | 251 (73.0%) | NA |
| N1 | 4 (1.1%) | NA |
| Unknown | 89 (25.9%) | NA |
| **M classfication** |  |  |
| M0 | 262 (76.2%) | NA |
| M1 | 4 (1.1%) | NA |
| Unknown | 78 (22.7) |  |
| **Tumor grade** |  |  |
| Grade 1 | 46 (13.4%) | NA |
| Grade 2 | 168 (48.8%) | NA |
| Grade 3 | 118 (34.3%) | NA |
| Grade 4 | 12 (3.5%) | NA |


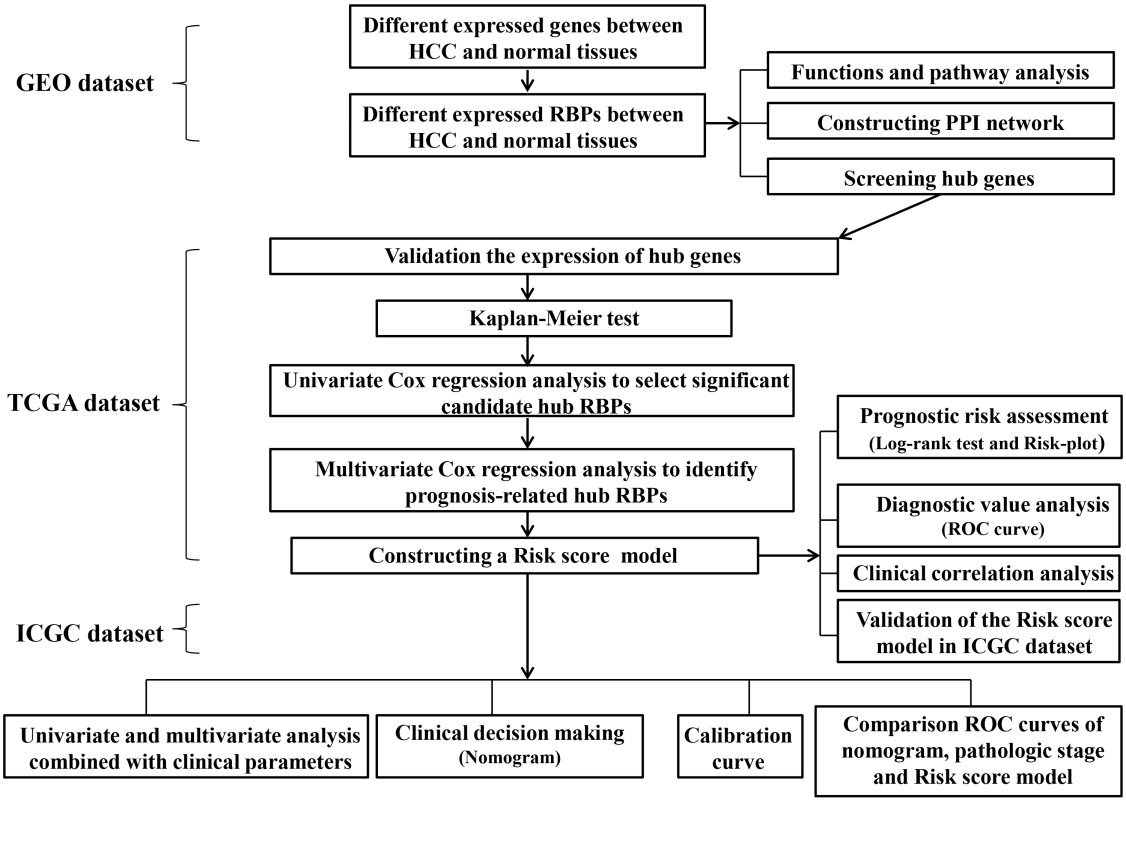


**Supplementary Figure 1. The flow chart of the overall analysis procedures**.


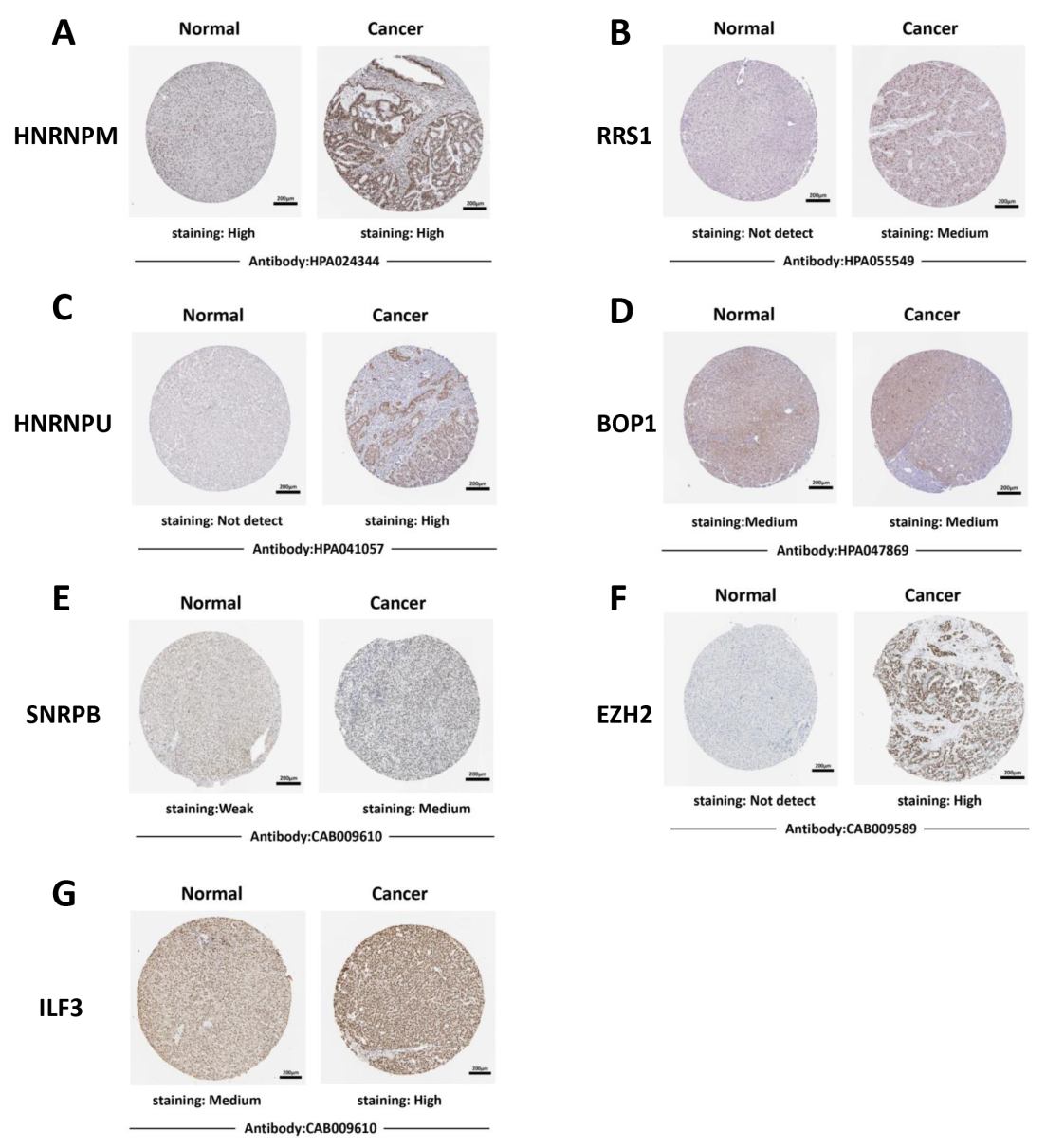


**Supplementary Figure 2. The representative immunohistochemistry image of hub RBPs in HCC tissues and adjacent normal tissues (Human Protein Atlas).** (A) HNRNPM; (B) RRS1; (C) HNRNPU; (D) BOP1; (E) SNRPB; (F) EZH2; (G) ILF3.


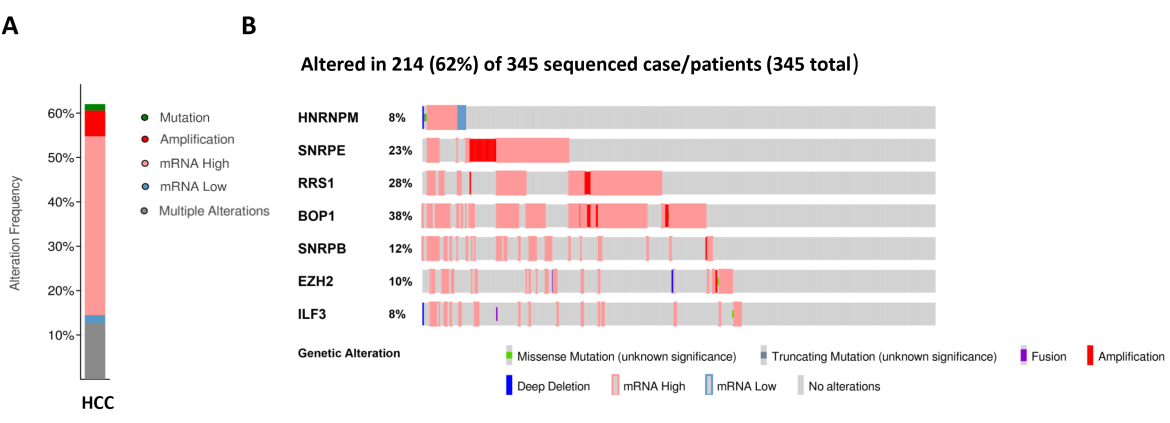


**Supplementary Figure 3**. **Mutations in prognosis-related RBP genes.** (A) Total alteration frequency; (B) Mutation frequency of each hub gene.


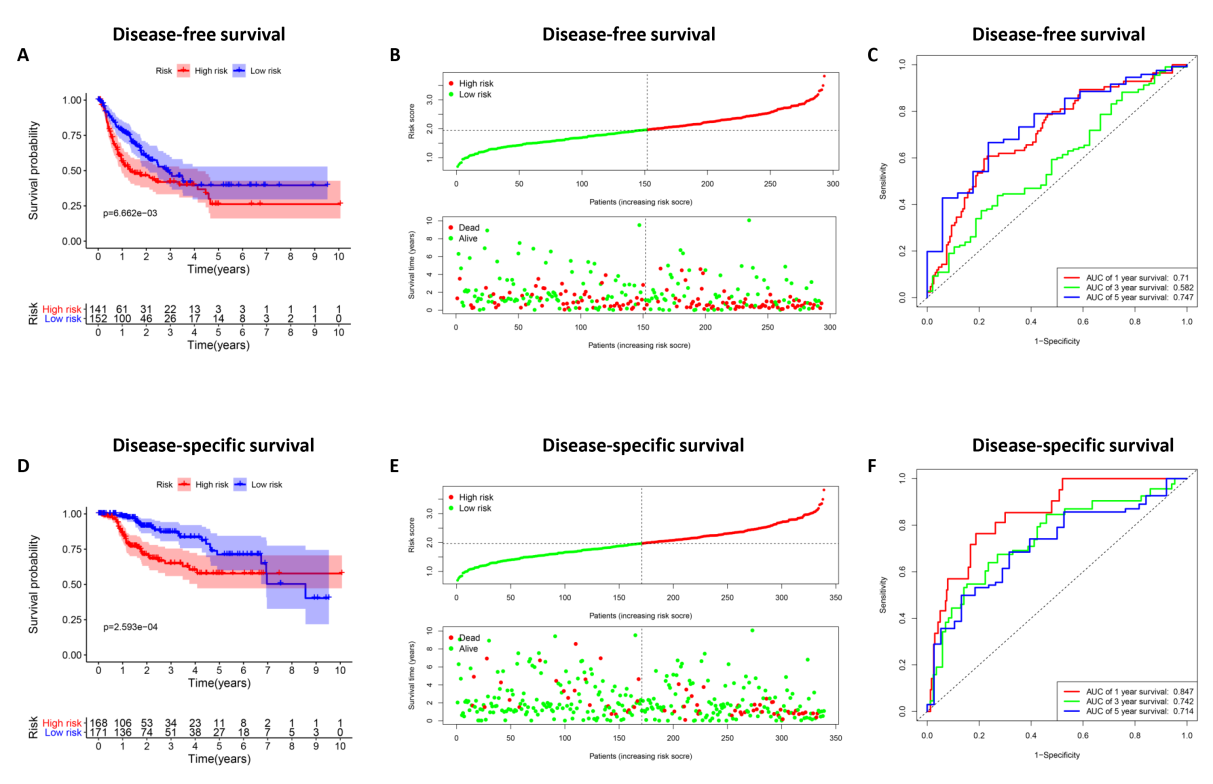


**Supplementary Figure 4**. **Risk score analysis of the prognostic model in the TCGA dataset.** (A) Disease-free survival analysis of high- and low-risk groups; (B) Risk score distribution and disease-free survival status of HCC patients; (C) ROC curves for predicting DFS based on risk score; (D) Disease-specific survival analysis of high- and low-risk groups; (E) Risk score distribution and disease-specific survival status of HCC patients; (F) ROC curves for predicting DSS based on risk score.


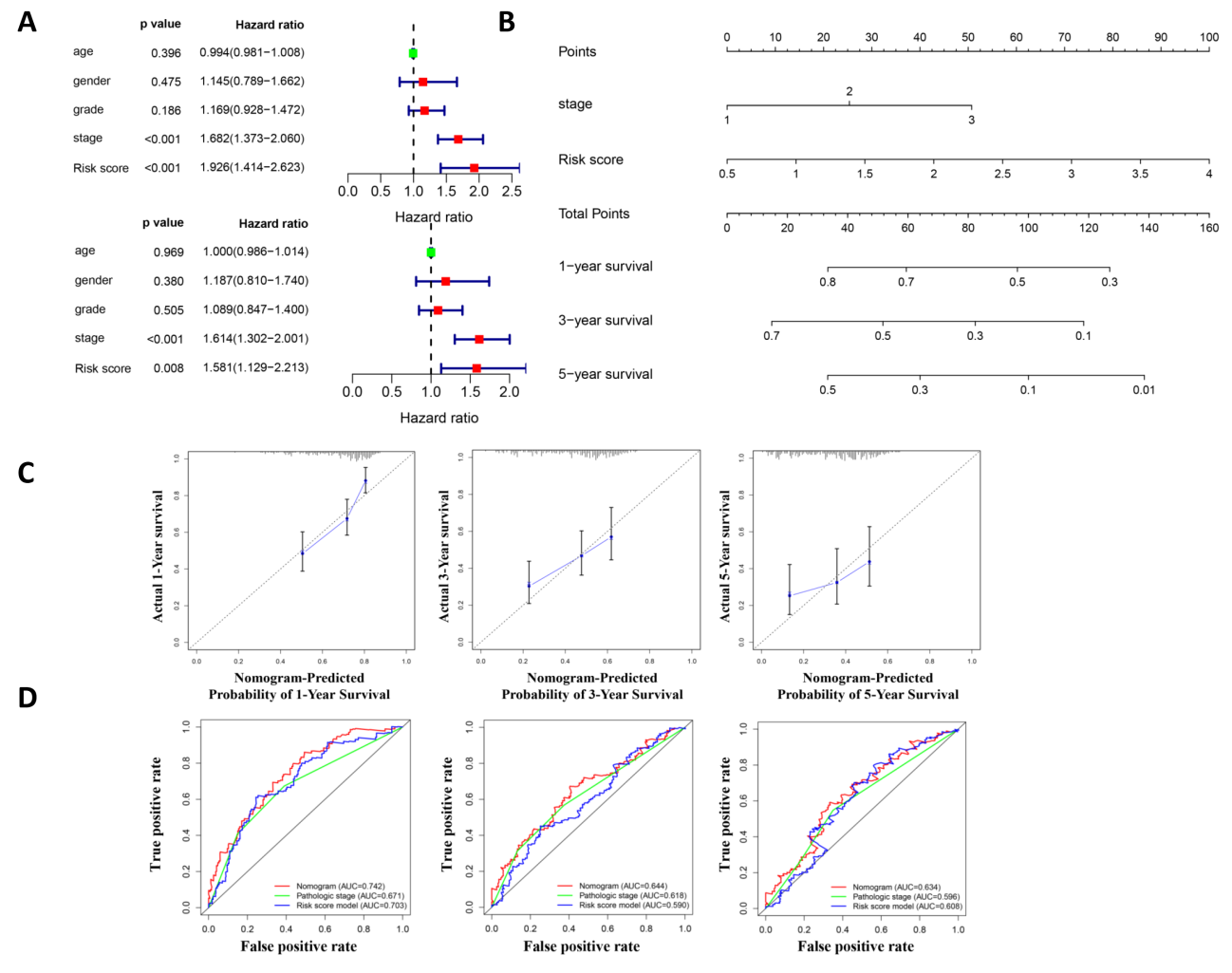


**Supplementary Figure 5. A nomogram for disease-free survival prediction for HCC patients.** (A) Univariate and multivariate Cox analyses for the prognostic model and clinical parameters; (B). A nomogram to predict DFS at 1 year, 3 years, and 5 years of patients with HCC; (C). Calibration plots showing the prediction of DFS at 1 year, 3 years, and 5 years by the nomogram (D). Comparison of time-dependent ROC curves among nomogram, clinical stage and the prognostic model.
